# The Effects of Cigarette Smoking on Steroidal Muscular Relaxants and Antibiotics Used: A Prospective Cohort Study

**DOI:** 10.3389/fphar.2021.573832

**Published:** 2021-04-26

**Authors:** Na Liu, Feng Wang, Qian Zhou, Minhuan Shen, Jing Shi, Xiaohua Zou

**Affiliations:** ^1^ Department of Anesthesiology, Guizhou Medical University, Guiyang, China; ^2^ Department of Anesthesiology, The Affiliated Hospital of Guizhou Medical University, Guiyang, China

**Keywords:** smoking-epidemiology, muscular relaxants, postoperative infection, general anesthesia, perioperation

## Abstract

**Background:** The impact of cigarette smoking on perianesthesia management is not clear elucidated. This paper studies the impact of long-term cigarette smoking on the dose-response of rocuronium and vecuronium used under general anesthesia and the type of antibiotics used after surgery.

**Methods:** We enrolled 240 participants from a teaching hospital in China in which finally enrolled in 221 participants. 106 participants have a history of long-term cigarette use and 115 participants without a history of smoking. All participants received general anesthesia for various surgeries, and rocuronium was used as the muscular relaxant. The primary outcome was the effective onset time of rocuronium after adjusting for its dose. The secondary outcomes included a recovery index and the time of muscle recovery changing from 25 to 75%.

**Results:** There was no measurable difference in the muscle relaxant onset time, duration of effectiveness, 75% recovery, recovery index, dose of opiates, anesthetics during surgery, or complication rate between smokers or non-smokers. However, the results showed a significant difference in antibiotic use between smokers and non-smokers (chi-squared = 13.695, *p* < 0.001), and a significant difference in the type of antibiotics used (chi-squared = 21.465, *p* = 0.003). Smokers had a significantly higher rate of cefathiamidine use.

**Conclusion:** Smoking cigarettes had no effect on muscle relaxants used under general anesthesia, but patients who had a history of smoking were more likely to receive antibiotics after surgery.

**Clinical Trial Registration:** http://www.chictr.org.cn/index.aspx, identifier ChiCTR-OIC-16009157.

## Introduction

The number of smokers has reached one billion globally, and China ranks first with over 300 million smokers. Smoking is a public health problem. It increases the incidence of adverse events and significantly affects postoperative recovery, these effects include addiction, priming for use of other addictive substances, reduced impulse control, deficits in attention and cognition, and mood disorders (Schmid et al., 2015). A number of reviews show that cigarette smoking is associated with increased peri-operative complications (Beckers and Camu, 1991; Rodrigo, 2000). It increases the risk of hospital mortality by 20% and the risk of major postoperative complications by 40% (Ruppert et al., 2018).

Rocuronium bromide is a non-depolarizing neuromuscular blocking drug (NMBD). It works by competitively binding to nicotinic acetylcholine receptors on motor endplates. It is intermediate-acting, rapid-onset, and works without cardiovascular side effects. It was introduced in clinical settings as a potentially ideal muscle relaxant (Özcengiz, 2005). The factors that affect onset time of muscle relaxants have been studied in recent two decades, and smoking was reported to be one of them (Dawson and Vestal, 1982). Nicotine in cigarettes is an alkaloid that has an agonist effect on nicotinic-cholinergic receptors. Small doses of nicotine (<100 μg/L) directly stimulate the neuromuscular junction (acetylcholine-like action) and facilitate transmission of impulses. Large doses (>100 μg/L) block transmission because of persistent depolarization (acute effect) or desensitization of the receptor site (chronic effect) (Taylor, 1992). Several clinical studies investigating the interactions between neuromuscular blocking agents and cigarette smoking have been reported, but whether smoking has substantial impact on neuromuscular blockade is controversial, as these studies have yielded contradictory results (Teiriä et al., 1996). We therefore launched a study investigating the possible effects of smoking on the onset time of rocuronium bromide through a prospective cohort study.

## Methods

### Study Design

The study was approved by the Medical Ethics Committee of the Affiliated Hospital of Guizhou Medical University (Application Number: 2016-57) and was registered at the Chinese Clinical Trial Registry (Registration Number: ChiCTR-OIC-16009157). All the subjects gave informed consent before participating in the study.

From June 2016 to October 2018, we recruited patients undergoing surgery in the affiliated hospital of Guizhou medical university. All patients received general anesthesia for various surgeries. The inclusion criteria were patients over 18 years of age, volunteer participation in the study, and signed informed consent. The exclusion criteria were: a prior history of surgery; significant abnormal renal and liver function (AST 40 > U/L, ALT 50 > U/L, CRE > 73 μmol/L, or BUN > 7.5 mmol/L); disorders of neuromuscular junction and skeletal muscle; water and electrolyte disorder of extracellular fluid and intracellular fluid; taking medications that affect neuromuscular junction; or aggressive tumors.

### Data Collection

We collected data at enrollment and each clinical encounter through standardized data collection forms, and the data were entered into electronic databases for analysis. Data collection included two parts: self-reported outcomes and intraoperative measurements. Self-reported outcomes included baseline information (including age, sex, etc.), as well as details of cigarette smoking and postoperative use of antibiotics.

### Intraoperative Measurements

After the contraction response of electrical stimulation was stable, the calibration was completed, and the muscle relaxation was continuously monitored by four series of stimuli under the same conditions. The onset time for rocuronium was defined as the time from the end of the injection of rocuronium to the maximum depression of T1. The clinical action time was defined as the TOF ratio rising from 0 to 25%, and the recovery index was designated as the TOF ratio rising from 25 to 75%. The muscle relaxation monitoring concluded when the above three indicators were completed. The skin temperature tested in nasopharynges was monitored and maintained at not less than 32°C during the operation, and normothermia was also maintained throughout the procedure. After preoxygenated, sequential intravenous midazolam (0.05 mg/kg), 1% long-chain propofol (2 mg/kg), sufentanil (0.3 μg/kg), rocuronium (0.6 mg/kg) for anesthesia induction was conducted with mechanical ventilation after tracheal intubation, micro pump 1% medium long-chain propofol (6–8 mg/kgh), remifentanil (8–10 μg/kgh) after tracheal intubation, mechanical ventilation tidal volume (VT) (8–10 ml/kg), respiratory rate (12–16 times/min), to maintain an end-tidal carbon dioxide partial pressure of 35–45 mmHg. Anesthesia was maintained using intravenous and the BIS (bispectral index) of the EEG (electroencephalogram) was monitored to maintain a BIS value between 40 and 60.

### Data Analysis

Preliminary analyses were performed to check the missing values in the dataset, fill in missing values through multiple imputations, categorize participants’ ages into five levels (18–31 years, 31–40 years, 41–50 years, 51–60 years, and >60 years), categorize BMI (Body Mass Index) into three levels (<25 kg/m^2^, 25–28 kg/m^2^, and >28 kg/m^2^), categorize the duration of smoking history into three levels (no smoke, <10 years, >10 years), categorize surgery types as major or minor, and transform postoperative complications into dichotomous variables (yes/no).

The first objective of the study was to detect differences between smokers and non-smokers in the effects of the muscle relaxant and in the postoperative use of antibiotics and opiates. Considering that the sample sizes of smokers and non-smokers were nearly equal, we used Fisher’s exact test to determine whether significant differences between the two cohorts existed in categorical outcomes (categorized age; categorized BMI; categorized smoke duration; proportion of patients quitting smoke; type of muscle relaxants; disease history; surgery type; usage of antibiotics; type of antibiotics, anesthetics, and opiates; and proportion of patients with complications). We also used independent *t* test to determine whether significant differences existed in continuous outcomes (PE_T_CO2, onset time of muscle relaxants, lasting time of muscle relaxants, 75% recovery, and recovery index). We further used a logistic regression model to determine the robustness of Fisher’s exact test and independent *t* test. We also studied the interaction between surgery type and the use of antibiotics, opiates, or anesthetics.

The second objective of the analysis was to determine the correlation of smoking frequency with use of muscle relaxant, antibiotics, opiates, and anesthetics. We used a linear regression model to find out whether the correlation was significant. We also used the model to find interaction between surgery type and the use of antibiotics, opiates, or anesthetics.

Coefficients of the logistic regression model and the linear regression model were calculated along with their corresponding 95% confidence intervals (95% CIs).

## Results

### Characteristics of Study Participants

**FIGURE 1:**
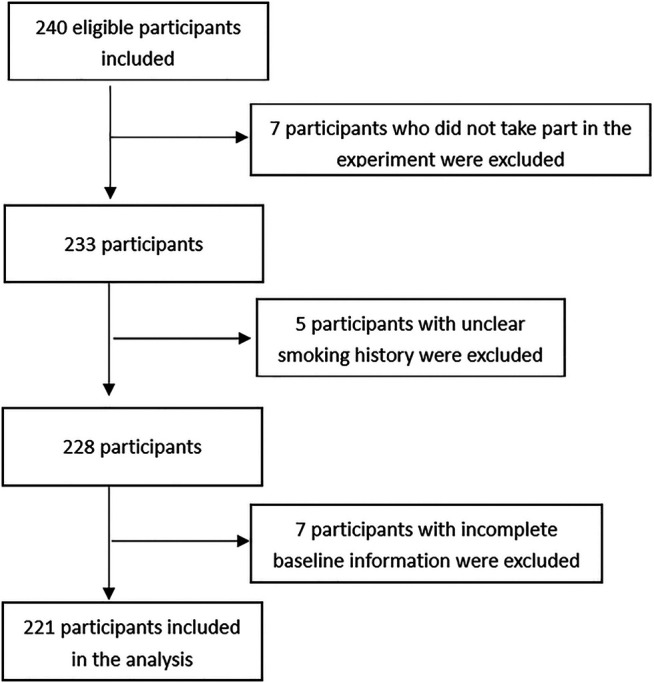
Describes selection process of the study participants.

Figure 1 describes the selection process for the participants in the study. A total of 240 participants were screened for participation. Five were excluded for unclear history of cigarette smoking; seven were excluded for unwillingness to participate in the study; and seven were excluded for incomplete baseline information. The cohort study finally enrolled 221 participants. Characteristics of these participants at enrollment were summarized, and comparisons between the smokers and non-smokers were made in Table 1. The participants had an age of 44.29 ± 11.02 years and a BMI of 23.03 ± 2.84. Smokers had a mean smoke period of 10.86 ± 13.1 years. Two (1.9%) of the smokers had quit smoking prior to surgery. Thirty-three (14.93%) of the participants had a history of diabetes, hypertension, or hepatitis. The participants received 15 (6.87%) major surgeries and 206 (93.23%) minor surgeries. In the process of surgical procedure, participants were maintained at a mean PE_T_CO2 of 36.07 ± 3.02, and they had a mean muscle relaxant onset time of 219.83 ± 60.04 s, a mean lasting effect of muscle relaxants of 2504.1 ± 799.22 s, a mean recovery index of 1165.91 ± 495.87 s, and a mean 75% recovery of 3672.84 ± 1015.99 s. A total of 138 (62.44%) participants were given postoperative antibiotic treatment. Five (2.26%) participants had postoperative complications.

**TABLE 1:** Characteristics of included studies.

Variables	Smoking (*n* = 106)	Non-smoking (*n* = 115)	*p* value
Age			
18–30	7 (6.6)	6 (5.2)	0.3
31–40	8 (7.5)	19 (16.5)	
41–50	26 (24.5)	27 (23.5)	
51–60	41 (38.7)	34 (29.6)	
>60	24 (22.7)	29 (25.2)	
BMI			
<25	2 (1.9)	11 (9.6)	0.04
25–28	6 (5.7)	4 (3.5)	
>28	98 (92.4)	100 (86.9)	
Smoke period			
<10 years	5 (4.7)	—	
≥10 years	101 (95.3)	—	
Quit smoking			
Yes	2 (1.9)	—	
No	104 (98.1)	—	
Muscle relaxants			
Rocuronium	68 (64)	93 (81)	0.008
Vecuronium	38 (36)	22 (19)	
History			
Hypertension	7 (6.6)	13 (11.3)	0.5
Diabetes	4 (3.8)	2 (1.7)	
Both hypertension and diabetes	2 (1.9)	2 (1.7)	
Others	1 (0.7)	2 (1.7)	
None	92 (87)	96 (83.5)	
Surgery type			
Major	8 (7.5)	7 (6.1)	0.9
Minor	98 (92.5)	108 (93.9)	
PETCO2	35.7 (2.8)	36.4 (3.2)	0.1
Muscle relaxant onset time (s)	217.1 (55.9)	222.8 (64.4)	0.5
Muscle relaxant lasting effect time (s)	2420 (738.2)	2582 (847.3)	0.1
75% recovery	3631 (1003.4)	3711 (1030.3)	0.6
Recover index	1206 (509.3)	1129 (482.5)	0.2
Usage of antibiotics			
Yes	80 (75.5)	58 (50.4)	<0.001
No	26 (24.5)	57 (49.6)	
Type of antibiotics			
cefamandole	2 (1.9)	7 (6.1)	0.0
cefathiamidine	22 (21.0)	10 (8.7)	
cefoperazone	3 (2.9)	1 (0.9)	
ceftizoxime	6 (5.7)	5 (4.3)	
cefuroxime	28 (26.7)	22 (19.1)	
Levofloxacin	9 (8.6)	6 (5.2)	
Piperacillin	10 (9.5)	7 (6.1)	
None	26 (24.8)	57 (49.6)	
Anesthetics			
Propofol	95 (89.6)	91 (79.1)	0.05
Other	11 (10.4)	24 (20.9)	
Opiates			
Sufentanil	103 (97.1)	112 (97.4)	1
Fentanyl	3 (2.9)	3 (2.6)	
Complications			
Yes	4 (3.8)	1 (0.9)	0.3
No	102 (96.2)	114 (99.1)	

### Impact of Smoking on Use of Muscle Relaxants, Antibiotics, Anesthetics and Opiates

**FIGURE 2:**
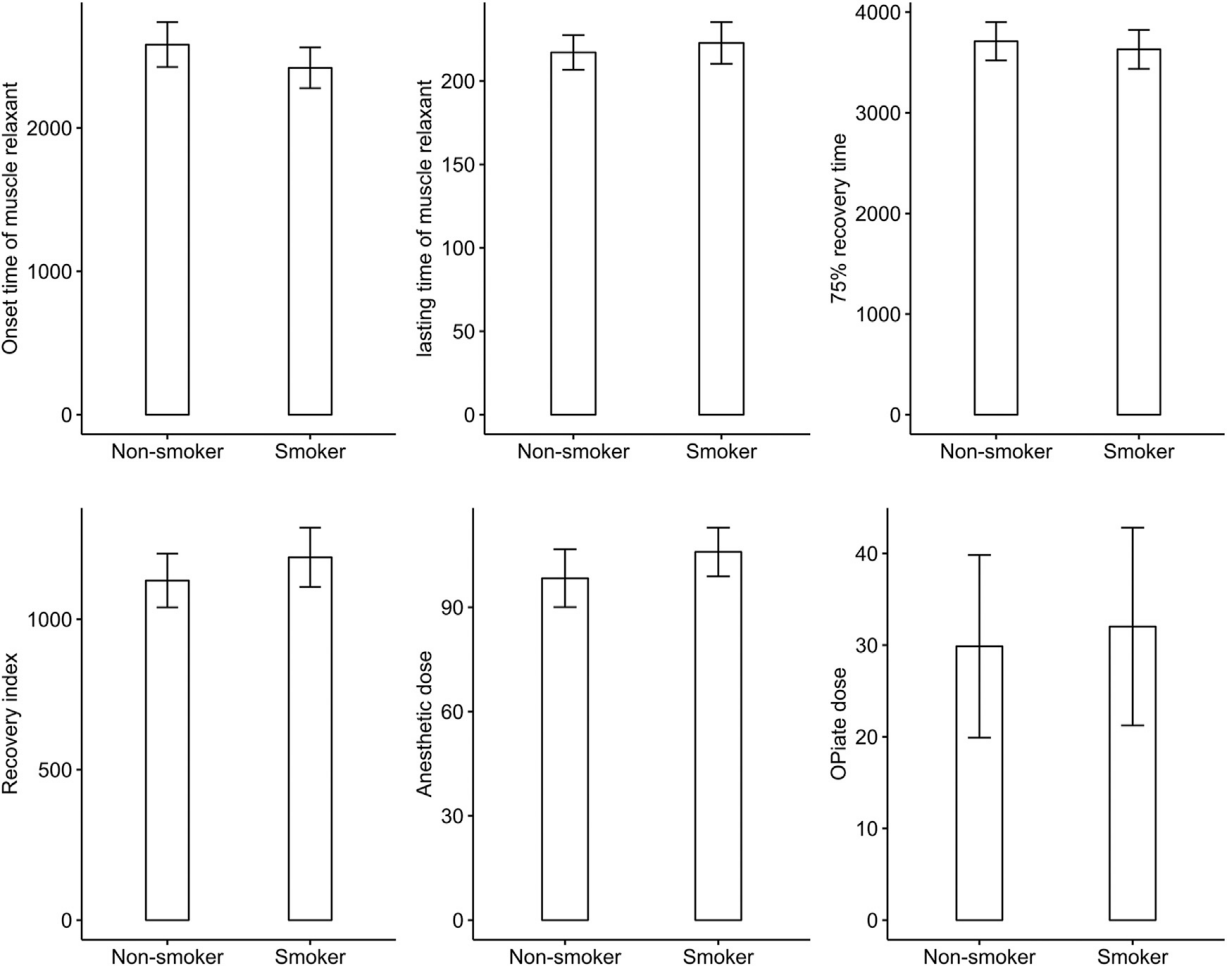
Comparison in anesthesia related parameters between smokers and non-smokers.

The result showed significant difference in antibiotic use between smokers and non-smokers (chi-squared = 13.695, *p* < 0.001) and significant difference in the type of antibiotics used (chi-squared = 21.465, *p* = 0.003). However, there was no difference in onset time of muscle relaxant, effective duration of musle relaxant, 75% recovery, recovery index, dosage of opiates and anesthetics during surgery, or complication rate between smokers or non-smokers. Logistic regression confirmed the significant difference in antibiotic use (odds ratio (OR) 2.87, 95% confidence interval (CI) 1.58 to 5.32; *p* < 0.001) between smokers and non-smokers, and showed that smokers had a significantly higher rate of cefathiamidine use (OR 7.92, 95% CI 1.50 to 62.48; *p* = 0.023). Table 2 shows the ORs of variables in the logistic regression analysis. Figure 2 shows the difference in onset time of muscle relaxant, effective duration of muscle relaxant, 75% recovery, recovery index, and dosage of opiates and anesthetics during surgery between smokers and non-smokers.

**TABLE 2:** Logistic regression analysis of the impact of cigarette smoking.

Variables	Coefficients	95% CI	*p* Value
Onset time	1.00	0.95 to 1.05	0.181
Lasting time	1.00	1.00 to 1.01	0.984
75% recovery	1.00	0.95 to 1.06	0.425
Recover index	1.00	0.95 to 1.05	0.975
Antibiotic use	2.87	1.58 to 5.32	0.960
Opiate dose	1.00	0.99 to 1.00	<0.001
Anesthetic dose	1.00	1.00 to 1.01	0.666
Complication	4.35	0.58 to 88.67	0.478
Cefathiamidine	7.92	1.50 to 62.48	0.024
Cefoperazone	9.08	0.68 to 266.53	0.124
Ceftizoxime	4.72	0.65 to 46.59	0.142
Cefuroxime	4.76	0.93 to 36.59	0.082
Levofloxacin	5.22	0.84 to 46.08	0.095
Piperacillin	4.67	0.80 to 39.49	0.109
Not use antibiotics	1.74	0.36 to 12.70	0.524

### Impact of Smoking History Duration on use of Muscle Relaxants, Antibiotics, Anesthetics and Opiates

We also found that smoking history duration was significantly correlated with antibiotic use (estimate 4.98, 95% CI 2.00 to 7.97; *p* = 0.001) (Table 3). However, smoking history duration was not significantly correlated with onset time of muscle relaxant, effective duration of muscle relaxant, 75% recovery, recovery index, dosage of opiates and anesthetics during surgery, or complication rate. Further analysis showed that a longer history of smoking was correlated with higher rate of cefathiamidine use (estimate 7.92, 95% CI 0.38 to 16.40; *p* = 0.04).

**TABLE 3:** Linear regression analysis of the impact of cigarette smoking.

Variables	Coefficients	95% CI	*p* value
Onset time	−0.10	−0.32 to 0.11	0.349
Lasting time	0.00	−0.03 to 0.02	0.868
75% recovery	0.10	−0.11 to 0.32	0.354
Recover index	−0.10	−0.31 to 0.12	0.371
Antibiotic use	4.98	2.00 to 7.97	0.001*
Opiate dose	0.00	−0.03 to 0.02	0.722
Anesthetic dose	0.01	−0.03 to 0.04	0.694
Complication	4.67	−4.75 to 14.09	0.330
Cefathiamidine	7.92	0.38 to 16.40	0.040*
Cefoperazone	9.08	−4.25 to 20.90	0.193
Ceftizoxime	4.72	−6.25 to 12.98	0.491
Cefuroxime	4.76	−3.62 to 12.18	0.287
Levofloxacin	5.22	−5.92 to 11.92	0.508
Piperacillin	4.67	−3.82 to 13.36	0.275
Not use antibiotics	1.74	−7.55 to 7.43	0.988

## Discussion

Our study found that cigarette smoking had no effect on the dosage of muscle relaxant during general anesthesia, and we found that cigarette smoking was associated with higher rate of antibiotic use postoperatively. One study showed that smokers appeared to require less atracurium than non-smokers (Kroeker et al., 1994). However, another study showed that smokers need more rocuronium to achieve the same muscle relaxation effect than non-smokers, and the investigators attributed this finding to high metabolic levels and changes in receptor levels in smokers (Rautoma and Svartling, 1998). These two studies showed contradictory results. Latorre and colleagues studied the effects of smoking on rocuronium in 20 smokers (Latorre et al., 1997). They found that onset and recovery times were similar between smokers and nonsmokers, which could be partly due to a longer period of refraining from smoking in patients, leading to very low nicotine blood concentrations without the proposed receptor-stimulating effect. Friedrich and colleagues found similar outcomes, which showed that smoking did not change the dose-requirements for rocuronium and had no effects on the onset time, degree of blocking effect, time to maximum blocking effect, duration 10%, and spontaneous recovery index during anesthesia (Pühringer et al., 2000). The sample size of previous studies was small (40 cases at most), which made it difficult to explain the problem clearly. In contrast, we determined the effect of long-term smoking on muscle relaxants under general anesthesia through large sample tests. In our study, duration of smoking history had no significant correlation with the onset time of muscle relaxant, the duration of the muscle relaxant's action, the 75% recovery rate, recovery index, the dosage of opioids and anesthetics during surgery, or the incidence of complications. This finding supports the viewpoint that smoking does not alter the dose-requirements for rocuronium and has no effects on the onset time (Latorre et al., 1997; Pühringer et al., 2000).

Literature showed that small doses of nicotine can increase the excitability of synapse acetylcholine, prolong the onset time, and weaken the effect of neuromuscular blockade. To the contrary, a large dose of nicotine would reduce the excitability of acetylcholine and would lead to the opposite results (Rodrigo, 2000; Sweeney and Grayling, 2009). However, the results of our study show that there is no difference between relaxation times and the fact of smoking.

Multiple studies have demonstrated that smoking increases the likelihood pulmonary complications (Bluman et al., 1998; Kaufmann et al., 2018). However, our results showed no difference between non-smokers and smokers. This could be explained by the fact that most of the procedures were minor surgeries, and the duration of operation was generally short. In addition, the proportion of antibiotics use was larger in smokers, which may have reduced the risk of postoperative complications.

We found significant difference in antibiotic use between smokers and non-smokers (chi-squared = 13.695, *p* < 0.001) and significant difference in the type of antibiotics used (chi-squared = 21.465, *p* = 0.003). This suggests that smoking patients might be more likely to suffer from postoperative infections and need more antibiotics than non-smokers. Active smoking is associated with respiratory complications, particularly bronchospasm and pneumonia (Ruppert et al., 2018). Meanwhile, the strong positive correlation between prevalence of COPD, emphysema, and lung fibrosis and prevalence of current smoking was expected among current and former smokers. Neuromuscular blockers as auxiliary drugs for general anesthesia are commonly used in tracheal intubation, which can cause respiratory muscle paralysis and affect respiratory function during and after surgery. These may also partly explain a higher rate of antibiotic use in participants with smoking habits. In addition, this finding may also suggest that using more antibiotics is an empirical choice that the doctor makes knowing that the patient is a smoker. More research is needed to clarify the mechanism of this finding.

Clindamycin and aminoglycoside antibiotics can enhance muscle relaxant function (Singh et al., 1978; Schulze et al., 1999). These antibiotics inhibit the release of acetylcholine from the motor nerve terminals by binding to calcium ions on the surface of presynaptic membranes (Pridgen, 1956). Therefore, doctors tend to use cephalosporin antibiotics without neuromuscular blocking effect, to avoid adverse neuromuscular blockade after surgery. For patients with a long history of smoking, prophylactic use of antibiotics before surgery may help to reduce the risk of pulmonary infection. However, further research needs to be done on the types of antibiotics, the timing of use, dosage, and course of treatment.

Our study had limitations. First, this study design was observational, so results should be treated with caution. We matched the smokers and non-smokers to reduce potential bias in the analysis of the primary outcome. Second, the smoking period and amount of cigarette use were based on the participants’ self-report. Although we asked the participants to confirm their reports, the study was still under the risk of outcome assessment bias.

## Conclusion

In summary, smoking does not alter the dose-requirements for rocuronium and has no effects on the onset time or the incidence of complications. It showed significant difference in antibiotic use between smokers and non-smokers and significant difference in the type of antibiotics used.

## Data Availability

The raw data supporting the conclusion of this article will be made available by the authors, without undue reservation.

## Abbreviations:

BIS, bispectral index; BMI, body mass index; CI, confidence interval; EEG, electroencephalogram; NMBD, non-depolarizing neuromuscular blocking drug; OR, odds ratio; TOF, train of four; VT, tidal volume.
